# What Does Social Support Sound Like? Challenges and Opportunities for Using Passive Episodic Audio Collection to Assess the Social Environment

**DOI:** 10.3389/fpubh.2021.633606

**Published:** 2021-03-29

**Authors:** Anubhuti Poudyal, Alastair van Heerden, Ashley Hagaman, Celia Islam, Ada Thapa, Sujen Man Maharjan, Prabin Byanjankar, Brandon A. Kohrt

**Affiliations:** ^1^Division of Global Mental Health, Department of Psychiatry and Behavioral Sciences, George Washington School of Medicine and Health Sciences, Washington, DC, United States; ^2^Human and Social Development, Human Sciences Research Council, Pietermaritzburg, South Africa; ^3^Medical Research Council/Wits Developmental Pathways for Health Research Unit, Department of Pediatrics, Faculty of Health Sciences, University of the Witwatersrand, Johannesburg, South Africa; ^4^Department of Social and Behavioral Sciences, Yale School of Public Health, Yale University, New Haven, CT, United States; ^5^Center for Methods in Implementation and Prevention Science, Yale School of Public Health, Yale University, New Haven, CT, United States; ^6^George Washington School of Medicine and Health Sciences, Washington, DC, United States; ^7^Transcultural Psychosocial Organization (TPO) Nepal, Kathmandu, Nepal

**Keywords:** digital health, passive data, depression, mental health, adolescent, adolescent mothers

## Abstract

**Background:** The social environment, comprised of social support, social burden, and quality of interactions, influences a range of health outcomes, including mental health. Passive audio data collection on mobile phones (e.g., episodic recording of the auditory environment without requiring any active input from the phone user) enables new opportunities to understand the social environment. We evaluated the use of passive audio collection on mobile phones as a window into the social environment while conducting a study of mental health among adolescent and young mothers in Nepal.

**Methods:** We enrolled 23 adolescent and young mothers who first participated in qualitative interviews to describe their social support and identify sounds potentially associated with that support. Then, episodic recordings were collected for 2 weeks from the mothers using an app to record 30 s of audio every 15 min from 4 A.M. to 9 P.M. Audio data were processed and classified using a pretrained model. Each classification category was accompanied by an estimated accuracy score. Manual validation of the machine-predicted speech and non-speech categories was done for accuracy.

**Results:** In qualitative interviews, mothers described a range of positive and negative social interactions and the sounds that accompanied these. Potential positive sounds included adult speech and laughter, infant babbling and laughter, and sounds from baby toys. Sounds characterizing negative stimuli included yelling, crying, screaming by adults and crying by infants. Sounds associated with social isolation included silence and TV or radio noises. Speech comprised 43% of all passively recorded audio clips (*n* = 7,725). Manual validation showed a 23% false positive rate and 62% false-negative rate for speech, demonstrating potential underestimation of speech exposure. Other common sounds were music and vehicular noises.

**Conclusions:** Passively capturing audio has the potential to improve understanding of the social environment. However, a pre-trained model had the limited accuracy for identifying speech and lacked categories allowing distinction between positive and negative social interactions. To improve the contribution of passive audio collection to understanding the social environment, future work should improve the accuracy of audio categorization, code for constellations of sounds, and combine audio with other smartphone data collection such as location and activity.

## Introduction

The social environment, including social support, social burden, and quality of interactions, is important for a range of health outcomes, including mental and behavioral health. Social support has been shown to be one of the most important factors for mental health outcomes (1). For example, in the case of maternal depression, social support is one of the most important predictors of severity and duration (2). Social isolation and loss of social group membership are considered as risk factors for postpartum depression (3, 4). Additionally, changes in perceived social support influence the course of depression (5). Women who have positive and affectionate social support constantly available are significantly less likely to experience postpartum depression than those who have it available sporadically (6). Overall, postpartum support has been reported to improve infant and maternal well-being (7). The transition of adolescent and young women to motherhood is often psychologically stressful, and social support facilitates a smooth transition and the emotional well-being for mothers (8). Social support reduces morbidity and mortality, exposure to health hazards, and psychological stress (9). Ultimately, social support is an important factor in reducing the incidence of postpartum depression and other health issues, particularly among adolescent and first-time mothers.

Investigators have used different methods to assess and measure social support among mothers. For example, functional social support refers to informational, emotional, instrumental and appraisal support, and structural support encompasses both formal (i.e., from doctors, nurses, and midwives) and informal (i.e., from husbands, mothers, fathers, siblings, etc.) support (9). Other methods include interactions between family and community members, satisfaction with support received, and specific types of support, such as child care (10). Another widely used framework parses social support into three-dimensions: emotional support, instrumental support, and informational support in the context of postpartum depression (6, 11, 12).

There are many challenges that come with assessing social support. Self-report measures are the dominant approach to quantify social support, but this is often unreliable and socially biased. There is a need for more objective approaches to understand social environments, identify risk factors, and target and track social domains for change over time. In addition, social support is a multi-dimensional topic that does not have one universally agreed upon definition and thus is difficult to quantify. This creates challenges when interpreting outcomes of interventions that specifically target social support as the hypothesized mechanism of action for recovery from maternal psychological distress (10).

Passive sensing data is increasingly being used in mental health research (13, 14). It refers to the capture of users' information without their active input while they go about their daily lives (15, 16). One of the commonly used methods is smartphone-based passive sensing data. The Global Positioning System (GPS) location, physical activity and movement, and amount of time that a device or app is used, such as Web-based social activity are a few examples of passive data collection (17, 18). Another form of passive sensing is episodic audio recording that can capture brief snippets of the audio environment (19). The audio recording can be helpful in identifying speech and non-speech audio stimuli around users that can be indicators of social support and interactions. Machine learning can be used to categorize these sounds. This helps to preserve confidentiality because transcriptions or listening directly to audio is not required. The output of machine learning can be used to quantify the timing and frequency of different types of audio to understand the social environment (20). Thus, passive audio data can provide an important and unique insight into a mother's social environment through continuous data collection.

To explore the potential for passive audio collection as a window onto the social environment, we conducted a two-part study. First, we conducted qualitative interviews with adolescent and young mothers about their social experiences to identify sounds potentially associated with social support. Second, we collected audio for 2 weeks on mobile phones and used machine learning to quantify the frequency of different types of sounds. We combined the qualitative and passive audio collection findings to identify opportunities and challenges for future research on social support in the home environment.

## Methods

### Setting

This current study was part of a broader initiative to improve mental health services in rural Nepal (21), including a special focus on use of technology to improve maternal mental health for adolescents (19). We conducted the study in Chitwan, a southern region of Nepal with a population of 579,984. Chitwan has an under 5 mortality rate of 38.6 per 1,000 and a literacy rate of 78.9% (22). Chitwan has been the center for district-wide scaling-up of community-based mental health services in Nepal as a result of which there is an established partnership with the local health systems (23). We conducted the study in seven health facilities selected on the basis of number of postnatal mothers visiting the facility and availability of mental health treatment. Data collection was undertaken between November 2018 and April 2019.

### Study Population and Sampling

We screened 782 mothers at health posts during infant immunization camps. Eligibility criteria included mothers between the ages of 15–25 years with infants younger than 12 months of age to participate in the study (*n* = 320). We consented the mothers to participate in the study before they completed a depression screening tool, the Patient Health Questionnaire (PHQ-9), to assess depression symptoms. PHQ-9 is a common, well-validated tool used to measure depression symptoms severity across 9 items, with each item having 3 response options, ranging the score from 0 to 27. It has been used in prior studies in Nepal and validated in primary care settings in Chitwan. We categorized mothers into three groups: low-likelihood of depression (referred to as “not depressed”), high-likelihood of depression (referred to as “depressed”), and mixed-probability. Not-depressed were mothers with a PHQ-9 total score below 7. Depressed were mothers with a PHQ-9 cut-off of 9 or above. The mixed group was a PHQ-9 score of 7 or 8. Among Nepali adults presenting to outpatient services, a cut-off of 9 has a sensitivity of 94% and specificity of 69%, positive predictive value (PPV) of 0.33 and negative predictive value (NPV) of 0.99 (24). For non-depressed, a cut-off of less of 7 has a sensitivity of 100%, specificity of 55%, PPV of 0.26, and NPV of 1.00. This means that an adult presenting to primary care centers with a score of below 7 has an extremely low probability of having depression (i.e., low probability of being a “false negative”). We excluded mothers with PHQ-9 scores of 7 and 8 from either group because of greater risk false positives.

Once the mother was screened for depression and consented, a study team member visited her home for family consent. For participants under the age of 18 years, we asked for a written parental permission form and assent form. The study was approved by the Nepal Health Research Council (#327/2018) and George Washington University's Institutional Review Board (#051845).

Among the mothers screened, 320 were 15–25 years old, had an infant under 12 months, and had a PHQ-9 score of either <7 or ≥9. There were 294 categorized as non-depressed (PHQ-9 < 7) and 26 categorized as depressed (PHQ-9 ≥ 9). We serially enrolled non-depressed mothers who consented with a target sample size of 25. Because of drop-outs, 23 mothers were the final non-depressed sample. Only non-depressed adolescent and young adult mothers (PHQ-9 < 7) were included in the analyses presented in the current paper in order to provide a reference point for understanding social support. Social support for depressed and non-depressed mothers can be different. Findings from the current study on feasibility of collecting passive audio data to understand social support of non-depressed mother can inform future comparisons between depressed and non-depressed mothers' social environment.

### Study Procedure and Data Collection

#### Qualitative Data

We conducted in-depth interviews (IDIs) and administered Day-in-life tool to qualitatively assess mother's social environment. We reached out to the mothers within 2–3 days of first contact at health posts to conduct IDIs. We also included field notes from each participant encounter in the data analysis. Field notes provided cultural and environmental description of mothers' surroundings. These also include reflection from two observation tools that were administered during the study period. Home Observation Measurement of the Environment (HOME) (25, 26) and Observation of Mother Child Interaction (OMCI) (27) were observation tools used to draw impressions of mother's surroundings and her interaction with the baby to complement the qualitative findings. HOME is a 45-item tool used to measure child's exposure to his/her environment. This tool consists of 6 substances—responsivity, acceptance, organization, learning materials, involvement, and variety, which are elicited by the combination of rater observations during home visit and direct elicitation of self-report by the parent. This tool has been used extensively in South Asia (27–29) and culturally adapted for Nepal. OMCI is an 18-item tool that documents maternal responsive behaviors (responsivity, emotional support, support for infant attention, and language stimulation). In this paper, we have explored items from these tools that are indicative of mothers' social environment and mother-child interaction.

Female research assistants used semi-structured interview guides to conduct IDIs that intended to be 30–45 min. Questions were asked to understand maternal experience for adolescent and young adult mothers, including their interaction with the child. Mothers were asked about their support system, social environment including social responsibilities. We asked about coping and help-seeking behavior, as well as negative social interactions. We included The Consolidate Criteria for Reporting Qualitative Studies (COREQ) checklist as a Supplementary File (30). Besides IDIs and field notes, we administered Day-in-life tool to understand an adolescent and young adult mother's typical day. We asked mothers what they did the day before and recorded the responses.

#### Passive Audio Data

Prior to the current study, we evaluated the acceptability and feasibility of passive data collection in low-resource settings (31). Based on this, we selected passive sensing modalities that would be feasible and contribute to improving maternal and child mental behavioral health. This included episodic audio recording on mobile phones. For this study, we used the Samsung J2 Ace smartphone to collect passive audio data. It was an affordable phone (US $160) popular in the study setting. Low-end mobile phones in Nepal generally cost US $70-$120. The smartphone selected for the study was slightly more expensive than commonly-used devices. In the study setting, most individuals already own mobile phones or have family members who own mobile phones. Additionally, Samsung J2 Ace phone is widely available for purchase within Nepal. It was also the cheapest option that could effectively run all the features and apps required for the study.

Mothers were provided with the phone for the duration of the study. They returned the smart phone after data collection ended. To collect the audio data, we installed our custom-built Electronic Behavior Monitoring app (EBM version 2.0). The EBM app passively collected audio data for 30 s every 15 min between 4 A.M. and 9 P.M. A folder (Namaste) was created automatically once the EBM app was installed on the mobile phone. All audio data were stored in the folder “Audio” inside the Namaste folder. For episodic audio recording, the microphone in the phone recorded 30s audio clips every 15 min and saved the recordings in an m4a format. Every week, a research assistant then collected the audio data from the mother's phone and uploaded it in a secured cloud database for analysis. The audio data was then processed for sound classification by a machine learning system. In our previous studies, we had produced a video to explain these data collection processes to potential study participants (31), including information on confidentiality management, such as deleting audio files (32). In the current data collection, mothers could delete audio files before research assistants uploaded the data off of the phones. Mothers were also instructed that they could turn off the phone at any point during the day to assure confidentiality or address other data collection concerns.

### Data Analysis

#### Qualitative Data

IDIs were conducted and transcribed in Nepali. Written Nepali transcripts were then translated into English using an established Nepali-English translation procedure and mental health glossary. A bilingual translator preserved culturally meaningful words (33). The interview transcripts were then combined with field notes and independently read by two researchers (AH, AP) to generate common themes. AH and AP then generated a preliminary codebook that was modified by AH, DL, and AP. The codebook was modified while obtaining intercoder agreement. The intercoder agreement of 0.70 was attained. Three researchers (AP, AH, and DL) then independently coded the interview transcripts in NVivo 12 (34). After coding, code summaries were written for each of the domains by two researchers (CI and AT) following a thematic approach (35, 36). Summaries were revised after discussion with several authors (AP, AT, CI, and BK). Table 1 shows the domains, themes and related codes that we explored in this study.

**Table 1:** Qualitative themes and related codes.

**Domain**	**Theme**	**Related codes**
Interaction	Adult interactions	Positive social interaction Negative social interactions
	Mother-child interaction	Positive mother-child interaction Negative mother-child interaction
Support	Emotional support	Mother's emotional support Positive emotions Negative emotions Coping/help seeking Isolation/No social interaction Trust
	Instrumental support	Support in household chores Childcare support General help Coping/help seeking
	Informational support	Informational support Coping/help seeking
Others	Recreational events	Positive social interactions Non-human interaction
	Social/religious events	Common social interactions Positive social interactions Negative social interactions
	Physical activities/exercise	Movement
	Sporting activities	Movement
	Stressful noises	Violence

#### Passive Audio Data

On average, about 40 raw audio files were generated each day. Files were downloaded from the device by research assistants and then uploaded to a secure server. Here the files were processed using a pre-trained machine learning model based on a Convolutional Neural Network architecture (VGGish) available as part of the AudioSet project (37). AudioSet, released in 2017 by Google, is a large-scale audio events dataset. It was constructed using millions of 10 second YouTube videos that were manually annotated. The dataset contains 632 audio event classes and is presented as a hierarchical graph of event categories (e.g., animal -> dog -> barking). The categories cover human and animal sounds, musical instruments and common environmental sounds. The files were processed by our machine learning model and the audio classification label with the highest probability score was saved as a single comma separated csv file for weekly audio clips. The .csv file had audio predictions along with time and date stamps used for analysis. These data were then cleaned to remove data of mothers who dropped out of the study and fix outliers introduced by some readings missing a valid date time value. Standard exploratory data analysis was performed on these data, including the calculation of measures of central tendency, range and missing value analysis. Plots and tables were produced on the full dataset (4 A.M. to 9 P.M.). We conducted a manual validation of 6,450 s of audio sounds. A research assistant listened to randomly selected audio clips to validate whether the machine-predicted speech and non-speech sounds were accurate.

## Results

### Qualitative Results

We assessed mothers' social environment to identify a) individuals that are present in mother's social environment, and b) speech and non-speech sounds that reflect mothers' interactions, support, and other social domains (Table 1). Codes in parenthesis correspond to the qualitative reference number for the quotes provided in Supplementary File 1. Table 2 consists of the demographic information of the mothers.

**Table 2:** Demographic characteristics of the participants.

**Total participant (*n* = 23)**	**N (%) or mean range**
**Mother's age**	**19.5 years (16–25)**
16–18	7 (30.43%)
19–21	11 (47.83%)
22–25	5 (21.74%)
**Caste**	
Brahman/chhetri	5 (21.74%)
Janajati	13 (56.52)
Dalit	5 (21.74%)
**Religion**	
Hindu	18 (78.26%)
Buddhist	3 (13.04%)
Christian	2 (8.70%)
**Education**	
Primary	3 (13.04%)
Secondary	17 (73.91%)
Higher secondary	3 (13.04%)
**Child sex**	
Female	12 (52.17%)
Male	11 (47.83%)
**Child age**	
1–7 months	17 (73.91%)
8–12 months	6 (26.09%)
**Number of children**	
First child	19 (82.60%)
**Participant occupation**	
Housewife	18 (78.26%)
Agriculture	3 (13.04%)
Business	1 (4.35%)
Day labor	1 (4.35%)

Based on the qualitative interviews, a typical day started at around 6 A.M. in the morning and would include either taking care of the baby, e.g., changing or breastfeeding, or household chores that included making morning tea for the family. Mid-morning usually included preparing meals for the family. Usually husbands in a nuclear family and older female family members, such as mothers-in-law, and sisters-in-law, took up infant's caretaking roles when mothers were busy with household chores. Mid-morning to noon usually included washing dishes after breakfast, washing clothes, and cleaning. In the afternoons, mothers usually rested with the baby and watched television or YouTube videos. This was also the time she interacted with female family members, neighbors, and friends; male household members were typically out of the house working or socializing during this time of day. However, female family members were present at the home throughout the day and interacted in the afternoon after household chores and around afternoon tea. Some mothers shared using their mobile phones to call distant family and friends during the afternoon. Evenings were much like mornings with mothers cooking and washing dishes until 7 or 8 P.M. A typical day would end with mothers putting the baby to sleep and spending time talking to her husband or family members. Mothers would then go to sleep with their infants, with co-sleeping being common. When it came to family members, mornings and evenings were the most social time where the family came together to eat meals.

### Social Interactions

In the qualitative interviews, mothers described their social interactions throughout the day. We coded the qualitative accounts for potential sources of audio stimuli that could be associated with presence and quality of these interactions.

#### Adult Positive and Negative Social Interactions

*Positive social interaction* was strongly related to mother's increased comfort and trust of family members. Adult conversations were often on matters related to childcare (SOC_01). Besides immediate family members, mothers also interacted with their neighbors. This interaction was usually centered around the babysitting or playing with the infant (SOC_02). Although non-family members were involved periodically in these roles, mothers spent most of their time interacting with immediate family members. *Negative social interactions* such as yelling and screaming were reported in households where husbands or family members had alcohol use problems:

*Participant [P]: Previously my husband used to drink alcohol because of that I had tension in my mind*.

Interviewer [I]: At that time, what types of thoughts used to come in your heart-mind?

*P: At that time, I didn't have anyone to share my problems. Mostly I remained alone. His father [husband] used to drink alcohol and yell at me all the time. He used to get angry with me because of that I was unhappy. And I used to think that all this was happening because of me. But now he doesn't drink alcohol. Previously he used to stay outside but nowadays he returns home*.

20-year-old mother with 3-month-old infant

In such households, besides increased negative interactions, there were limited positive adult sounds (laughter, adult conversations), since mothers expressed limiting their conversations even when the family members were sober. Mothers were also likely to turn their mobile phones off when there were arguments at home.

Therefore, audio stimuli that can predict positive social interactions include adult conversations, gender-specific conversations, and laughter while stimuli that can predict negative social interactions include adult yelling, screaming, and crying.

#### Mother-Child Positive and Negative Interactions

Most *positive mother—child interactions* mentioned were of mother playing with the infant (SOC_04). Mothers expressed joy and satisfaction when they played with their baby with toys and rattles and heard their baby laughing or babbling:

*I: In your opinion, what does your baby want from you? Like love, talking with him etc*.

*P: He needs love. Someone to talk with him, friends etc*.

I: How do you know that your baby needs all these things?

*P: He starts to cry when I leave him inside the room, starts laughing and enjoying when someone remains with him and talks with him. Due to these types of behaviors, I came to know that he wants someone to talk with him*.

22-year-old mother with 12-month-old infant

Besides direct interaction, mothers enjoyed taking pictures and videos of the baby. *Negative mother-child interaction* often remained unreported in recorded interviews because mothers feared they would be considered bad mothers if they said they beat or yelled at their children. These incidents were reported in observational tool assessments and field notes. Through observational tool assessments, HOME and OMCI, we also observed mother's speech (items 2–8), physical punishment (items 12, 16), and hostile speech (items 14, 15, 17, 18) as sounds present in the social environment. Similarly, OMCI also gave information on mother positive and negative speech (items 1–6, 8–11), along with positive and negative infant reaction (items 14–16), such as laughing, babbling, squealing, when with mothers.

Therefore, adult sounds like mother's speech, laughter in conjugation with child babbling or child laughter can be an indication of positive-mother child interaction. Negative mother—child interaction can include audio stimuli such as mother screaming and crying along with child crying and screaming. Non-speech sounds such as baby rattles and toys along with adult sounds and infant-related sounds can be an indication of mother-child interaction.

### Support

We explored this domain as supportive or non-supportive outcomes of social interactions. Unlike interaction, we studied support beyond the mere presence or absence of certain sounds in mother's environment. In this domain, we explored constellations of sounds, or combination of sounds that predicted supportive or burdensome social environment for the mother.

#### Emotional Support and Burden

Family members were the primary source of emotional support, especially husbands. Mothers generally considered “talking about her feelings” or “sharing problems with her family” an important coping mechanism (SUPP_01). Integrating mothers' conversations with family members and positive sounds such as laughter and singing can predict mothers' emotional environment. Besides family, neighbors and health care professionals were other important sources of emotional support (SUPP_02). The role of neighbors and health care professionals was even more prominent when the mother had strained family relationships and less emotional support from family members. Frequent conversations of mothers with members outside of the family, infant sounds (laughing, crying, squealing) along with adult sounds of non-family members, and positive/negative adult sounds (laughter, crying) could be indications of mothers seeking support outside of the family members.

I: How did you get rid from those [mental health] problems?

*P: My family members supported me and they suggested that I take care of my baby*.

I: And you got rid of those problems?

*P: Umm. All my relatives and family members supported and gave suggestions. They suggested that I have to love my baby; I have to breastfed my baby on time*.

19-year-old mother with 5-month-old infant

I: Why do you feel comfortable asking help from your landlord aunt [neighbor]?

*P: She feels very happy to play with my daughter and she always asks me [if she can take care of the baby] when I have to go somewhere. So, I feel easier to ask her for help*.

17-year-old mother with 3-month-old infant

Another important prediction of mother's emotional burden can be social isolation. Isolation or no social interaction was prevalent among mothers who were generally worried about the future of their children, either because of financial difficulty or family problems. If family members were the primary source of stress, mothers were not comfortable sharing these thoughts with their family members. This emotional burden also led to mothers feeling negatively about the baby, especially when the baby did not sleep or cried a lot. A complete lack of human speech for a prolonged period of time can be an indication of social isolation (SUPP_03, SUPP_04). Presence of machine-generated speech sounds such as TV/radio without human speech for a prolonged period of time could be another important indication of isolation.

*P: Because of the birth of my baby, I had different types of [negative] thoughts like how should I provide care to her and at that time I wanted to stay alone, didn't want to eat anything etc*.

I: What types of activities did you do to decrease those problems?

*P: I wanted to stay alone, walk, or listen to songs*.

19-year-old mother with 5-month-old infant

Hence, audio stimuli that predict emotional support or burden can be positive and negative adult speech sounds (conversations, laughter, singing, crying, yelling), frequent adult speech sounds (family members, non-family members), along with complete absence of speech sounds, or presence of machine-generated speech sounds (such as TV, radio) without adult speech sounds.

#### Instrumental Support

Two kinds of instrumental support were important—childcare support and support in household chores. In nuclear families, husbands were strongly involved in providing instrumental support to their wives, but older female members such as mother-in-law and sister-in-law in joint families were equally engaged (SUPP_05). To understand instrumental support better, audio data can be used to predict mother's speech sounds along with that of the family members within the house, especially around infant sounds (child crying, child laughing, babbling) indicating childcare support, or household chores sounds (washing, doing dishes, sweeping) indicating support in household chores.

I: When did you feel difficulty?

*P: Some days after delivering the baby, the baby did not feel well and we had to admit this baby to the hospital. At that time, I had to face many difficulties*.

I: At that time, who helped you?

*P: I got support mostly from my family members. My mother-in-law cooked food, washed clothes, and the baby's father [my husband] also supported me a lot*.

21-year-old mother with 5-month-old infant

Much like emotional support, instrumental support was hindered when the mother did not have good relationship with the family members. The family relationship and role of family in instrumental care were critical mostly because most mothers only trusted family members to take care of the baby in her absence. Strong family involvement was even more critical in case of young mothers who were generally inexperienced in childcare and had to learn it either experientially or through demonstration by other family members. If either of these did not happen, mothers had significant instrumental burden to take care of the baby. If the family members did not support her in household chores, she had to take care of her child on top of her regular household after childbirth. The lack of instrumental support meant mother was constantly involved in taking care of the child as well as household chores.

We can record the lack of instrumental care by assessing the amount of household chores sounds (cooking, washing, and sweeping) and infant-related sounds (breastfeeding, child bathing, oil massaging, child laughing, babbling, and crying) in a typical day, especially a comparison of these sounds alone and in combination with adult speech sounds, which might indicate presence of other adults. Another way to get a snapshot of mother's social environment is through observation tools. For instance, HOME item 20 asks about childcare support provided by one of the three regular substitutes, and HOME item 41 explores the role of the father in providing at least some form of childcare daily.

Therefore, instrumental support or burden can be understood through positive and negative speech sounds (adult conversations, adult laughter, adult yelling, and crying) in conjugation with child sounds (child babbling, child laughter, oil massaging, and child bathing), or household sounds (washing, sweeping, and cooking).

#### Informational Support

Given adolescent and young adult mothers' lack of experience in childcare, informational support is another important supportive outcome of social interactions. Family members were crucial in providing instrumental support both in young mothers' maternal home (father, mother, and sister) and husband's home (mother-in-law, father-in-law, and sister-in-law). Young mothers generally needed support in feeding the baby, bathing and oil massaging. Culturally in Nepal, oil massaging is a big part of infant's growth and development, and one in which mother needed the most support. Demonstration (SUPP_06) by an older female member (mother-in-law, mother, sister, sister-in-law) along with informational support (SUPP_07, SUPP_08) was significant in supporting young mothers. An important way to capture the informational support could be adult conversations within household in conjugation with infant-related sounds (child bathing, breastfeeding, oil massaging). Few mothers said they learned to take care of the baby without any external informational support from family members, friends, or health care professionals (SUPP_09).

“*When I asked her about the challenges of being mother, she shared that she didn't feel any difficulty. At first, she used to feel difficult to take care of her baby i.e. bathing her baby but later on when her mother, sister and mother in-law taught her then she felt easy.”*

Field notes (18-year-old mother with 2-month-old baby)

*P: In the hospital, sister [Nurse] told me more things [about childcare] than my family members. I got more information from her. She told me how to provide proper care to a newly born baby*.

21-year-old mother with 4-month-old infant

Almost all mothers had some form of informational support, primarily from family members, but also from health care professionals and neighbors. Therefore, we can have a better understanding of mother's information support system outside of the family if we collect passive audio data that captures mother's conversations with non-family members, especially conversations with health care professionals.

Therefore, positive adult speech sounds (adult conversations, laughter) and frequent adult speech sounds (family members, non-family members) along with infant-related sounds (child bathing, breastfeeding, oil massaging) can indicate informational support.

### Other Social Domains

We explored other common domains of mothers' social environment for better understanding of speech and non-speech noises that constitute these phenomena.

#### Recreation

Young mothers were very comfortable using mobile phones to watch YouTube videos or use social media, mostly Facebook and IMO (a messaging application popular in the study area) (OTH_01). Additionally, mothers often recorded videos on their phones or transferred and played songs/videos from their friends or family member's phones for recreation. Smartphones were important to the mothers for recreation, either to listen to songs or engage in conversations with family and friends through messaging applications (OTH_02). Mothers also used messaging app and smartphones to talk to husbands who were away for employment. Mothers also had audio alerts on their phones when they received instant messages.

I: Usually what do you use on your mobile [phone]? Which applications do you use?

*P: IMO [Messaging app]*.

I: Oh, usually you use IMO on your mobile, and do you feel comfortable using that application on your mobile?

*P: Um, I use IMO when I have time in the evening, and usually once or twice in a week when my brothers call me*.

20-year-old mother with 4-month-old infant

#### Social or Religious Events

Social and religious events were a big part of mothers' environment. These events constituted of religious functions, characterized by bells, singing bowl, religious instruments or social functions like marriage, with musical instruments and wedding bands. In both the events, frequent and multiple adult human speech can indicate presence of many people in the surrounding.

#### Physical and Sporting Activities

Movement or physical activity was a big part of adolescent and young mothers' lives. Bicycling and walking around the neighborhood were pretty common when running errands. Some mothers mentioned walking recreationally, to reduce mental stress. Movement was highly restricted for the first few months after childbirth for both physical and cultural reasons. Culturally, new mothers are often asked to stay home for at least the first 6 months of childbirth to facilitate breastfeeding and reduce the risk of infections. Environmental noises suggestive of outdoor activities include vehicles, birds, domestic animals, whistles, and activity sounds (bicycling and walking sounds).

#### Stressful Events

Some mothers described that violence was also a part of their lives. The mothers reporting violence were most likely to attribute this to husbands. For these mothers, there was a long-term exposure to violence often starting early in the marriage, throughout pregnancy and post-childbirth. In terms of her social environment, speech (adults arguing, crying, screaming, yelling) and non-speech (objects thrashing, hitting) could provide some indication of violence and disruptive family behaviors. Frequency and amount of these exposures in mother's environment can provide stronger evidence of stressful events in her surroundings.

“*I did this last follow up interview in the participant's maternal home. She went there to celebrate a festival and she felt comfortable having a conversation with me because she was away from her husband's home. At that time, she shared about her problems with her husband's family, problems that she felt difficult sharing in her husband's home. Because of this I came to know various things about her family that she tried to hide from me during my previous visits and interviews [at her husband's house]. She shared all these things openly without any hesitations. Her husband used to scold her. And he used to beat her during pregnancy. She tried to stay away from her husband when he got angry.”*

Field notes; 16-year-old mother with 4-month-old infant

### Pilot Passive Audio Data Collection

In our pilot study, we wanted to determine if we could successfully collect passive audio data from adolescent and young adult mothers and whether this data could give preliminary insights into the mothers' social environment. We successfully collected 14 days of audio data using the EBM app installed (with their knowledge) on the mobile phones provided to them. We were able to use a pre-trained machine learning model to predict sound categories (speech, vehicle, music etc.). Finally, we were able to determine the most common sounds in mothers' social environment along with the distribution of these sounds throughout the day.

A total of 319 categories of sounds were identified in 7,725 audio clips collected from 23 participants each recruited to participate for 14 days. About 42.7% (*n* = 2,318) of the sounds were human speech sounds, 35.4% were music sounds, and 6.3% were vehicle sounds. A manual validation of the 6,450 s of speech sounds showed 73% of the machine-predicted speech sounds were true speech sounds (Table 3). Of all the machine predicted non-speech sounds 62% were speech sounds.

**Table 3:** Sounds accuracy (*n* = 215 audio clips).

	**True speech**	**True non-speech**
Speech	157 (73.0%)	58 (27.0%)
Non-speech	133 (61.9%)	82 (38.1%)

**Figure 1:**
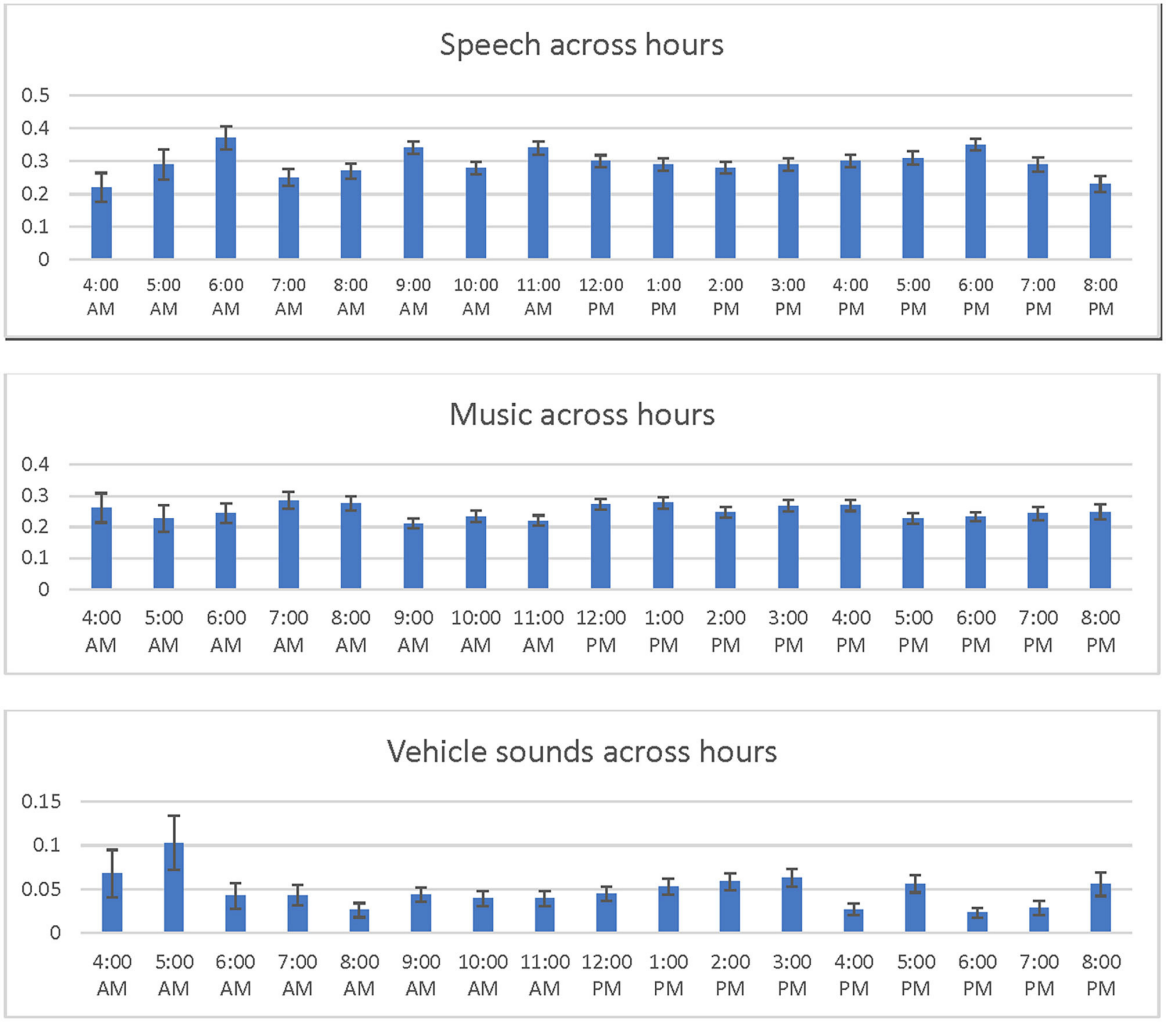
Common sounds throughout the day. This shows the average distribution of speech, music, and vehicle sounds from 4 A.M. to 9 P.M. each day.

Machine-predicted speech sounds were uniformly distributed throughout the day (Figure 1). We also assessed music and vehicle noises throughout the day. Music was consistently present throughout the day (range 21–29%), while vehicle noises were more prevalent in the morning (range 4–10%) than during the day (range 4–6%), or evening (range 3–6%). Table 4 shows the 10 most commonly predicted sounds.

**Table 4:** Ten most common sound categories.

	**Frequency**	**Percent**
Speech	2,318	42.67
Music	1,920	35.34
Vehicle	340	6.26
Organ	184	3.39
Rail transport	143	2.63
Tubular bells	131	2.41
Fire	125	2.30
Bus	101	1.86
Car	89	1.64
Insect	82	1.51
Total	5,433	100

Finally, we also tried collecting audio stimuli that could be strong predictors of the social environment. Since we used YouTube trained machine learning, these nuanced sounds were not detected frequently, and could not therefore be used as strong predictors of social environment. Table 5 shows the audio stimuli predicted by YouTube trained machine learning. These audio stimuli were not manually validated.

**Table 5:** Other audio social sounds and their prediction frequency.

**Other audio social sounds**	**Frequency**	**Percentage**
Child speech	9	0.12
Male singing	7	0.09
Whispering	7	0.09
Whimper	4	0.05
Babbling	3	0.04
Female singing	3	0.04
Female speech	3	0.04
Child singing	2	0.03
Children playing	2	0.03
Male speech	2	0.03
Children shouting	1	0.01

## Discussion

We qualitatively assessed mother's social environment and identified audio stimuli that could give us a better picture of her social environment. We then collected audio data from adolescent and young adult mothers for 14 days and used YouTube-trained machine learning to predict audio sounds. Qualitatively, we verified that mother's social environment mostly comprised of family members, and she was always surrounded by husband, mother-in-law, father-in-law, sister-in-law, and brother-in-law providing supportive roles especially during first few months of childbirth. Speech sounds for adult (laughter, adult speech) and child sounds (child laughter, babbling) were common around mother's social environment. Most common non-speech sounds included mothers doing household chores (washing, cooking) or childcare activities (bathing, oil massaging the baby). Mothers also engaged in recreational activities (watching YouTube videos, TV, listening to radio) frequently. Through passive audio data analysis, we identified speech as the most frequently detected sound, followed by music and vehicles.

Based on our qualitative findings and passive audio collection, we propose a matrix for what types of social interactions and activities could potentially be captured through the audio environment (see Table 6). Speech and non-speech sounds such as laughter, adult speech, TV noises, and social functions could be predictors of positive interactions. Similarly, crying, yelling, along with sounds of beating, thrashing, or object breaking showed negative interactions. A prolonged presence of any of these positive or negative sounds for a given mother could help predict her household environment. Studies have previously captured the number of conversations and duration to successfully determine social interaction (38). Passive audio data have been used to estimate the number of conversations a person engages in, the duration of the conversation, and the time the individual speaks within the conversation along with speaking rate and variation in pitch (39, 40) to understand social isolation (41).

**Table 6:** Mother's social environment and potential speech and non-speech sounds.

**Domain**	**Theme**	**Speech sounds**	**Non-speech sounds**	**Additional assessment**
Interaction	Positive mother-child interaction	Baby talk, child laughter, mother laughter, motherese, mother singing, lullaby	Sound of rattles, toys	Proximity beacon, ^*^HOME, ^**^OMCI, application use [videos and photos of baby]
	Negative mother-child interaction	Adult yelling, adult crying, child screaming, child crying	Physical violence (slapping, thrashing)	Proximity beacon, HOME, OMCI, call logs, application use [photos and videos of baby]
	Positive adult interactions	Adult laughter, adult speech	TV noises, movie theater, music, social functions noise,	Passive data collection tool in family members' phones, call logs
	Negative adult interactions	Adult yelling, adult crying	Physical violence (slapping, thrashing), objects breaking, thrashing, loud noises of objects dropping	Proximity beacon, passive data collection tool in family members' phones, call logs
Support vs. burden	Emotional support or burden	Gender-specific speech detection, Person-specific audio (voice recognition)	TV and radio noises, songs, total silence	HOME, daily diary elicitation Maternal social support scale, sleep monitoring
	Instrumental support or burden	Family members' voice recognition, voice recognition (family vs. outside), lack of human speech, mother doing household chores alone (lack of interaction in integration during household chores)	Mother household chores (e.g., washing utensils) along with positive human interaction (laughter, talking), constant household chores noises (washing clothes, washing dishes)	Proximity beacon, OMCI, HOME, daily diary elicitation, ^***^NIH toolbox adult social relationship scales
	Informational support	Talking	TV, radio	Application usage, browser history
Others	Recreational activities	Individual singing, humming, multiple people singing	Music, TV, YouTube (videos)	Application usage, browser history
	Social events	Adult speech, laughter	Music, instruments (organ, piano, band music)	GPS, daily diary elicitation
	Religious events	Prayers, multiple people singing (Nepali: *bhajan*)	Puja bells/vedic chanting	GPS, daily diary elicitation
	Physical activities/exercise	Adult speech, adult laughter, crowd noises	Outdoor noises, vehicular noises, foot tapping, running/walking noises, sweeping, washing, bicycling	Accelerometer, GPS
	Sporting activities	Crowd noises, cheering	Whistles, bicycling	Accelerometer, GPS
	Stressful noises	Constant loud human speech, persistent human noises for a long duration	Non-stop transportation noise, horns	Pollution indicators environment–environmental noise, Accelerometer, sleep monitoring

* *Home Observation for Measurement of the Environment*.

** *Observation of Mother-Child Interaction*.

*** *National Institutes of Health*.

Based on our initial experiences, social support is a more complex concept and requires understanding beyond presence and absence of audio stimuli. We suggest an analysis of “constellations of audio sounds” around the mother to give us a better picture of her social support i.e., drawing social environment conclusions based on grouping of speech and/or non-speech sounds. This would require integration of audio clips from a given time frame that predict mother's social support. For instance, “instrumental support” could be categorized when the following combination is present: (a) audio of mother laughing, (b) adult female speech of a family member, and (c) audio of washing dishes. Understanding the nature of social support would also require gender differentiation (male vs. female speech). Non-speech sounds, both positive and negative, can help determine support and burden in mother's environment. Additional tools such as daily diary elicitation, social support scale (42, 43), and sleep monitoring (44, 45) can give additional assessment of her support system.

In our matrix, we have suggested additional data collection tools that can further validate the findings from passive audio. For example, application and call logs have also been used to determine communication (46). In case of mother-child interaction, we suggest using additional observation tools such as HOME (27–29) and OMCI (47–49) which have been used in multi-cultural settings to assess mother and child's social environment, interactions and support. Finally, additional methods of passive data collection, such as Bluetooth beacons attached to child's clothing, can help determine the time mother and child spend together (19, 31).

Finally, other social noises such as social events, recreational activities, physical activities, and stressful events can be good predictors of overall social environment. We can understand the time and modes of recreation for the mothers if we capture passive audio data across sounds of YouTube videos, and messaging apps. Additionally, passive audio features such as application and call logs can also help us understand her environment. In addition to passive audio data, Global Positioning System (GPS) (50) and accelerometer (40, 46) have been successfully used in studies to determine movement and activity. Passive audio data can be critical in identifying domestic violence. Intimate partner violence (IPV) has been associated with depressive symptoms (51, 52) but remains highly underreported in Nepal (53). Passive audio data can give an invaluable insight into stressful events of mother's lives predicting domestic violence.

Additional assessment methods such as Ecological Momentary Assessment (EMA) (54) have triggers tied to hearing certain sounds which asks if a sound is accurate when heard in the environment. Such assessment methods can validate passive audio clips. Mood tracking tied to speech and non-speech sounds is another efficient method of understanding mother's mood associated with particular audio stimuli (46). Studies have also explored alternative coding approach for culturally diverse audio (55).

Additional validation tools in combination with passive audio data can provide important insight into mothers' lives especially under domains like mother-child interaction and domestic violence, which are difficult to assess, especially in low- and middle- income settings (48, 56). Studies focused on passive sensing data often use additional validation measures to ensure accuracy (13). We suggest similar approach to studying domains such as interaction and support. Passive audio data can generate evidence on the quantity of sounds, but qualitative assessments and/or multiple passive data methods can provide a more reliable picture of the social environment. Additionally, it is important to consider the measures of audio data that might be most meaningful. While domains such as interactions can be assessed by the presence or absence of human speech, domains like support might require total number and frequency of speech, time-specific contact, predictability of interactions and amount of audio stimuli observed/expected. Passive audio data when combined with appropriate qualitative and observational tools such as daily diary elicitation, ecological momentary assessment, HOME, Quality of mother-child interaction, and additional passive data collection methods like Global Positioning System (GPS) (50), Bluetooth beacons (19, 31), sleep pattern detection (45), accelerometer (40, 46), including recording call logs (46) and application usages (57), and device activity (58) can provide unique and innovative insight into mother's social environment.

## Limitations

The major limitation of our current study was the accuracy of the pre-trained model. One of the major challenges to accurately collecting passive sensing data was inaccuracy of YouTube trained machine learning. The social and cultural environment of adolescent and young adult mothers are not universally consistent, so prior to the passive audio data collection, it is integral to record and train some of these culturally relevant sounds from the study setting to train the machine. Similarly, a strong prediction model to distinguish individual-specific audio stimuli such as adult vs. child speech, female vs. male speech, and sounds such as adult vs. child laughing, can predict positive/negative adult conversations along with positive and negative mother-child interaction.

Moreover, social support and social interaction cannot fully be understood without better models to identify positive and negative sounds, such as polite vs. hostile conversation, yelling or laughing sounds. These audio stimuli are culturally sensitive and must be recorded prior to the study for better prediction of the environment. It is equally integral to distinguish between machine-generated speech sounds such as TV/radio and adult speech sounds, mostly when studying social isolation, when continuous exposure to machine-generated speech sounds can have very different impact than that of adult speech sounds. Another important consideration for future studies should be more contextual information on mobile phone usage in the setting. In our study, some mothers carried phones more consistently than others. In some instances, other family members used the mobile phone along with the mother. So, we need to consider that the audio captured might be different from mother's actual experience. A thorough understanding of mother's usage patterns and social factors (such as cell phone sharing) needs to be considered when drawing meaningful conclusion.

Another limitation of our study is the sampling technique which could impact the generalizability of the findings. We used purposive sampling, therefore our findings might not be representative of the population. We had to constantly modify how we explained the study to the mothers and their families as we learned how best to clarify the technology use. This led to a mix in data capture, with low data collection at the beginning and more toward the end of the study. We only collected audio data between 4 P.M. and 9 P.M., so we could have missed important speech and non-speech sounds outside of the study period. With the advancement of cellular networks in rural Nepal, the use of Android-based smartphones is getting more popular. We anticipate more acceptance of mhealth initiatives such as these in the future. A better understanding of average adolescent and young mother's lifestyle and cultural barriers to her use of technology will be integral in successful implementation of mobile health and passive data studies in Nepal.

## Conclusion

Using passive audio data to capture the auditory environment of adolescent and young mothers gives a unique opportunity to improve our understanding of their social environment. Besides information on total speech and non-speech exposure, it can help assess quality and frequency of these sounds. Although current methods have limited comprehensive distinction between positive and negative social interactions, nuanced sounds like child laughter, child crying, adult laughter, or distinction between machine (TV, radio) vs. adult speech, a stronger model trained using culturally appropriate sounds can provide prediction of mother's auditory environment. We recommend a strong machine learning model combined with techniques such as coding of constellation of sounds, validation with observation, and qualitative tools for stronger indication of mother's environment Combining additional passive sensing data such as location and activity can also be integral in understanding mother's social support and interactions.

## Data Availability Statement

The raw data supporting the conclusions of this article will be made available by the authors, without undue reservation.

## Ethics Statement

The studies involving human participants were reviewed and approved by Ethical approval was received from the Nepal Health Research Council (#327/2018) and George Washington University Institutional Review Board (#051845). Written informed consent to participate in this study was provided by the participants' legal guardian/next of kin.

## Author Contributions

AP and BK drafted the manuscript. AP and AHa conducted the qualitative data analysis. AHa supervised the qualitative data collection and analysis. AHe developed the EBM app. AHe and PB developed the StandStrong app. AHe and AP conducted the quantitative data analysis. SM supervised data collection and onsite study implementation. BK, AHe, and AHa conceptualized the study and study design. All authors revised the manuscript.

## Conflict of Interest

The authors declare that the research was conducted in the absence of any commercial or financial relationships that could be construed as a potential conflict of interest.

## Abbreviations

COREQ: Consolidate Criteria for Reporting Qualitative Studies
EBM: Electronic Behavior Monitoring
EMA: Ecological Momentary Assessment
GPS: Global Positioning System
HOME: Home Observation for Measurement of the Environment
IDI: In-depth Interviews
OMCI: Observation of Mother-Child Interaction
PHQ-9: Patient Health Questionnaire – 9.
